# Combined Brain–Heart Imaging in Takotsubo Syndrome: Towards a Holistic Patient Assessment

**DOI:** 10.3390/jcm13102991

**Published:** 2024-05-19

**Authors:** George Markousis-Mavrogenis, Alessia Pepe, Flora Bacopoulou, Amalia Lupi, Emilio Quaia, George P. Chrousos, Sophie I. Mavrogeni

**Affiliations:** 1 University Research Institute of Maternal and Child Health and Precision Medicine, and UNESCO Chair in Adolescent Health Care, Medical School, National and Kapodistrian University of Athens, ‘Aghia Sophia’ Children’s Hospital, 11527 Athens, Greece; georgemm32@gmail.com (G.M.-M.); bacopouf@hotmail.com (F.B.); chrousge@med.uoa.gr (G.P.C.); 2 Institute of Radiology, Department of Medicine, University of Padova, 35128 Padova, Italy; alessia.pepe@unipd.it (A.P.); amalia.lupi@phd.unipd.it (A.L.); emilio.quaia@unipd.it (E.Q.); 3 Onassis Cardiac Surgery Center, 17674 Athens, Greece

**Keywords:** takotsubo syndrome, brain, heart

## Abstract

Takotsubo syndrome (TTS) is a type of cardiomyopathy usually precipitated by either emotional or physical stress and potentially leading to reversible heart failure. There is emerging evidence indicating an interaction between the brain and the heart in patients with TTS. Nevertheless, these new insights are not reflected in the current clinical approach to TTS. The application of novel and existing imaging modalities for the evaluation of brain–heart interactions is an interesting approach that could potentially augment diagnostic and prognostic yield, as well as improve our pathophysiologic understanding in the context of TTS. In this opinion piece, we discuss the evidence supporting a brain–heart interaction in patients with TTS and discuss how a combined evaluation of brain–heart interactions could potentially be implemented.

## 1. Introduction

Takotsubo syndrome (TTS) is a type of cardiomyopathy characterized by transient regional systolic dysfunction, dilatation, and edema, primarily affecting the left ventricular (LV) apex and/or midventricular myocardium. TTS is an acute disease entity that can ultimately lead to reversible heart failure, with LV dysfunction usually subsiding after a period of days to weeks [1].

Patients with TTS may present with acute chest pain, dyspnea, syncope, or ventricular arrhythmia/cardiogenic shock in severe cases [2]. Diffuse T-wave inversion, sometimes preceded by ST-segment elevation and mild cardiac enzyme elevation may be identified. Characteristically, a coronary angiography will not reveal any culprit coronary lesions that could explain the clinical presentation [2]. However, according to more recent evidence, coronary artery disease (CAD) may also co-exist with TTS and these patients may present with the entire spectrum of acute coronary syndrome [3]. TTS typically occurs in post-menopausal women, with 10% of patients being men in some case series. Emotional or physical stress often precedes the clinical presentation, although no stressful stimulus can be identified in approximately a third of cases [1,2].

Considerable scientific evidence supports the existence of brain–heart interactions in the context of TTS. The Comorbidity Frequency in Takotsubo Syndrome (COUNTS) study documented the presence of emotional and physical stress as triggering factors in 39% and 35% of 1109 TTS cases, respectively [4]. Furthermore, a considerable proportion of patients had comorbid conditions, including mental disorders (24%), pulmonary vascular disease (15%), nervous system disease (7%, most commonly subarachnoid hemorrhage), and trauma (1%) [4]. Furthermore, a history of chronic anxiety was identified in some women before the development of TTS [5].

**Figure 1:**
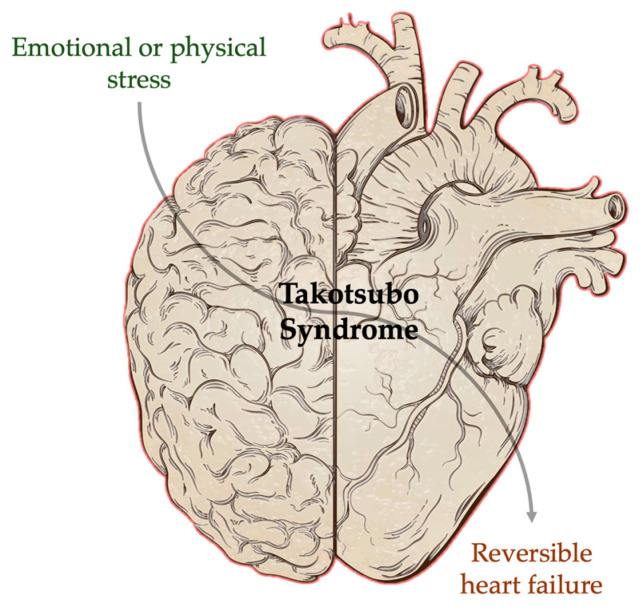
Graphical representation of brain–heart interaction in TTS.

Despite the aforementioned evidence, there is currently no established role for the concomitant evaluation of the brain and the heart in patients with TTS. In this Opinion Paper, we summarize scientific evidence regarding the interaction between the brain and the heart in the context of TTS and argue for the utility of non-invasive brain–heart imaging for the evaluation of these patients (Figure 1).

## 2. Mechanisms of Brain–Heart Interaction in Takotsubo Syndrome

Stress is a physiologic response regulated by the central nervous system (CNS) and the endocrine system. In the CNS, the main anatomic areas involved in the regulation of the stress response are the limbic system, neocortex, spinal cord, reticular formation, and brainstem [6]. Exposure to a stressor leads to excitation of the locus coeruleus–adrenomedullin axis and hypothalamus–pituitary–adrenocortical (HPA) axis [7]. The locus coeruleus is the central site of noradrenergic neurons in the brain stem and its activation leads to the secretion of norepinephrine by adrenal medullary chromaffin cells, which stimulates the HPA axis [8,9]. Activation of the HPA axis results in adrenocortical production and release of cortisol, which further promotes the synthesis of adrenaline and noradrenaline in the adrenal medulla. The hypothalamus is physically connected to the amygdala and hippocampus, which can also stimulate the HPA axis [10].

The heart is also intimately innervated by the autonomic nervous system [11]. The sympathetic nerves descend through the spinal cord, then synapse at the sympathetic chain. The postganglionic neurons interact with the myocardium and the epicardial coronary arteries. The sympathetic nerve endings then activate the *α* and *β* postsynaptic adrenergic receptors through the release of noradrenaline [10]. The heart also receives parasympathetic innervation [11].

Interestingly, numerous studies have demonstrated the possibility of brain–heart interactions in patients with TTS. In a study of cerebral blood flow (CBF), using ^99^Tc-single photon emission computed tomography (SPECT), CBF in the brainstem, hippocampus, and basal ganglia was found to be significantly increased in patients with TTS. In contrast, blood flow to the prefrontal cortex was reduced [12]. Other authors reported anatomical and physiological alterations in brain regions controlling emotional and autonomic functions in patients with TTS [13]. Another study identified significantly reduced connectivity in the left amygdala, both hippocampi, left superior temporal pole, and right putamen of patients with TTS [14]. Furthermore, reduced connectivity in the limbic systems of patients with TTS was identified by other authors [15]. However, perhaps the most compelling evidence of a brain–heart interaction in the context of TTS can be derived from the retrospective analysis of brain imaging findings prior to the onset of TTS. In individuals with or without TTS who underwent previous brain ^18^F-fluorodeoxyglucose (FDG)-PET/CT imaging for other indications, those with TTS retrospectively had higher baseline amygdala activity independent of common risk factors for TTS. Additionally, among the patients that eventually developed TTS, those with higher amygdala activity were more likely to develop TTS earlier compared with patients that had lower amygdala activity [16]. Thus, there is currently ample evidence demonstrating the neurological changes in patients that have or go on to develop TTS. As such, it is conceivable that a simultaneous evaluation of the brain and heart may provide additive value in the diagnostic workup of patients with TTS and may even aid in the identification of high-risk individuals before disease development. We have previously reported findings from combined brain–heart MRI in patients with various autoimmune rheumatic diseases [17]. A similar approach could, for instance, also be used in patients with TTS.

## 3. Imaging Brain–Heart Interactions in Takotsubo Syndrome

A combined evaluation of the brain and the heart can be achieved using clinically available imaging modalities such as molecular/nuclear imaging (as mentioned in the previous section) and magnetic resonance imaging (MRI).

### 3.1. Molecular/Nuclear Imaging

Molecular/nuclear imaging allows for the evaluation of various pathophysiologic phenomena, including metabolic, perfusion, and inflammatory alterations, as well as the degree of sympathetic activation.

#### 3.1.1. Metabolic and Sympathetic Activity Imaging

The glucose analog [^18^F]-fluorodeoxyglucose (^18^F-FDG) is taken up by the brain and myocardium and is ideal for the evaluation of metabolic alterations in both organs [18]. Cardiac positron emission tomography (PET) using ^18^F-FDG has shown abnormal glucose metabolism in patients with TTS [19]. Cardiac fatty acid metabolism can also be assessed using ^123^I-beta-methy-iodophenyl pentadecanoic acid (^123^I-BMIPP) SPECT and has been found to be affected in patients with acute TTS [20]. Sympathetic activity can be evaluated using ^123^I-meta-iodobenzylguanidine (MIBG). In the subacute phase of TTS, adrenergic hyperactivity can still detected as decreased uptake/increased washout of MIBG due to regional lesions in sympathetic neuronal activity [19]. In the acute and subacute phases of TTS, similar alterations of MIBG and FDG uptake are also detected. Delayed recovery of both cardiac glucose metabolism and sympathetic innervation have been documented in patients with TTS and their identification allows for the diagnosis of TTS in patients, even after a few months following the previous stressful event [19].

#### 3.1.2. Inflammation Imaging

The use of ^18^F-FDG for inflammation imaging in the brain and heart is complicated by non-specific uptake in cortical and myocardial tissue. Other clinically used tracers target chemokine/cytokine receptors, adhesion molecules, matrix-degrading proteins and/or other metabolic mechanisms involved in the regulation of immune responses [21]. The 18 kD translocator protein (TSPO), which is produced in activated immune cells, is an interesting imaging target for the assessment of inflammation, and various radiotracers targeting TSPO have been used clinically to date [22]. There is currently a paucity of evidence regarding brain/heart inflammation imaging in patients with TTS and this could be the subject of future research endeavours.

#### 3.1.3. Perfusion and Blood Flow Imaging

Nuclear imaging can also be employed for the evaluation of cerebral and myocardial perfusion and blood flow. Applications of nuclear imaging for myocardial perfusion are well known and have been previously discussed in detail [23]. As stated previously, CBF assessed using ^99^Tc- SPECT was also found to be decreased in the brainstem, hippocampus, and basal ganglia and was significantly increased in the prefrontal cortex of patients with TTS [12]. Additional applications in patients with TTS have yet to be investigated.

### 3.2. Magnetic Resonance Imaging (MRI)

Magnetic resonance imaging (MRI) is a non-invasive diagnostic modality that does not use radiation and can provide valuable and highly reproducible information regarding function/perfusion/tissue characterization in the brain and heart. MRI can be used for illustrating brain–heart interactions in patients with TTS, notably in the same examination. Our group has previously published results from a combined brain–heart evaluation in patients with autoimmune rheumatic diseases [17]. A similar approach could conceivably be applied in TTS, although its clinical utility remains to be determined.

#### 3.2.1. Brain MRI

MRI is a powerful diagnostic tool for evaluating the brain in various diseases. In patients with TTS during the acute phase of the disease, a brain MRI showed a smaller cortical surface area and greater cortical thickness compared with matched controls. Similarly, the gray matter centers were smaller, except for the thalamus and insula, which were larger [24]. In another study, hypoconnectivity in central brain regions correlated with autonomic function and the regulation of the limbic system in patients with TTS [15]. Furthermore, as mentioned previously, there are anatomical and physiological alterations in the brain regions controlling the emotional and autonomic (both sympathetic and parasympathetic) functions in patients with TTS [13]. In the acute phase of TTS, volumetric and functional changes in frontal regions and the central autonomic network (i.e., insula, anterior cingulate cortex, and amygdala) were noted [25]. Additionally, compared to healthy controls and patients with acute myocardial infarction, limbic responses to aversive visual stimuli were attenuated during the acute phase of TTS [26]. The main brain MRI findings in patients with TTS are summarized in Table 1.

#### 3.2.2. Cardiovascular Magnetic Resonance

Cardiovascular magnetic resonance (CMR) is the diagnostic gold standard for the evaluation of cardiac function, perfusion, and tissue characterization [27]. In recent years, the clinical use of CMR has significantly expanded and can currently be viewed as a “non-invasive biopsy” for myocarditis, non-ischemic cardiomyopathies, and various infiltrative diseases [28].

CMR can visualize the typical regional wall motion abnormalities characteristic of TTS, such as apical or mid-ventricular ballooning. In addition, CMR can characterize the presence of myocardial oedema and fibrosis. The presence of edema in the absence of irreversible tissue injury (late gadolinium enhancement) typifies TTS, although sometimes a positive late gadolinium enhancement could be present, especially in patients with mild cardiac enzyme elevation. CMR may also facilitate the diagnosis of other suspected cardiac conditions (myocardial infarction, myocarditis) [27].

During the acute phase of TTS, CMR is recommended to differentiate TTS from myocardial infarction with non-obstructed coronary arteries (MINOCA) or myocarditis. Furthermore, in the post-acute TTS phase, CMR is recommended in all patients with persistent electrocardiographic and/or regional wall motion abnormalities in the echocardiography performed [19].

### 3.3. Combined Nuclear and Magnetic Resonance Evaluation of Brain–Heart Interactions

To our knowledge, there are currently no studies that have investigated the use of combined nuclear and MRI studies in patients with TTS. However, the two imaging modalities can offer complementary information, as the nuclear approach may have a non-specific pattern, while MIBG can, for instance, provide information regarding adrenergic activity that cannot be provided by MRI. Furthermore, a complementary role could be provided in patients who might have contraindications for one of the two imaging modalities (e.g., renal insufficiency or implanted metallic clips/non-MRI conditional devices for MRI, high exposure to radiation in young or pregnant patients for nuclear imaging, etc.). The increased availability of combined PET–MRI systems could potentially allow for the enhanced evaluation of brain–heart interactions in patients with TTS [29,30].

## 4. Conclusions

To conclude, TTS is in essence a cardio-neuro-endocrine disease and it is conceivable that it should also be evaluated as such, instead of pursuing an isolated evaluation of the heart. A combined evaluation of the nervous/neuroendocrine system and the heart may have significant clinical implications by allowing for a more holistic patient approach, as well as a better understanding of the interplays between the various organ systems in the pathophysiology of TTS. Although such combined evaluation has not yet seen widespread implementation in the clinical setting, we would argue that there is already sufficient evidence to at least motivate its study in patients with TTS. However, the exact benefits in terms of hard outcomes or improved risk prediction at the patient level remain to be investigated.

## Figures and Tables

**Table 1:** Primary brain MRI findings in patients with Takotsubo Syndrome compared with healthy controls [24].

Increased cortical thickness and reduced surface area Larger volume of Thalamus and Insula Functional hyperconnectivity in pathways controlling cardiac autonomic functions No difference in white matter hyperintensities

## Data Availability

Not applicable.
